# Clinical Presentation and Management of Endometriosis-Related Hemorrhagic Ascites: A Case Report and Systematic Review of the Literature

**DOI:** 10.7759/cureus.15828

**Published:** 2021-06-22

**Authors:** Mareesol Chan-Tiopianco, Wei-Ting Chao, Patrick R Ching, Ling-Yu Jiang, Peng-Hui Wang, Yi-Jen Chen

**Affiliations:** 1 Division of Obstetrics and Gynecology, San Lazaro Hospital, Manila, PHL; 2 Department of Obstetrics and Gynecology, ManilaMed - Medical Center Manila, Manila, PHL; 3 Department of Obstetrics and Gynecology, Taipei Veterans General Hospital, Taipei, TWN; 4 Department of Medicine, University of Maryland Medical Center Midtown Campus, Baltimore, USA; 5 Institute of Clinical Medicine, National Yang-Ming University, Taipei, TWN

**Keywords:** ascites, bloody ascites, endometriosis, hemorrhage, hemorrhagic ascites

## Abstract

This study aims to analyze the patient profile and presentation of endometriosis-related hemorrhagic ascites and review its management to raise awareness among gynecologists and improve treatment strategies. We present a case report and engage in a systematic review involving human cases of histologically proven endometriosis with hemorrhagic ascites. Keywords were searched in PubMed/MEDLINE, Cochrane Library, EMBASE, and Ovid Discovery databases from inception until December 2018. Studies that did not include a description of ascites or histopathologic results confirming endometriosis or those that involved patients with other conditions that may contribute to ascites were excluded.

The review yielded 73 articles describing 84 premenopausal women with histologically proven endometriosis-related hemorrhagic ascites. Of note, 83% (65/78) of the patients were nulliparous and 69.35% (43/62) were of African descent. The most common chief complaint was abdominal enlargement (58.33%, 49/84) but a host of other symptoms were also reported. Pleural effusion was reported in 32.14% (27/84), and elevated CA-125 was seen in 74.42% (32/43). The majority (64.29%, 54/84) of the patients underwent laparotomy, and an increasing trend of minimally invasive surgical approaches (p<0.001) and fertility-sparing techniques (p<0.001) was observed. The mean ascites volume was 4228.27 mL (SD: 2625.66). Moderate to severe endometriosis was seen in 97.44% (76/78) of cases. The majority of the patients who received medical treatment were given gonadotropin-releasing hormone (GnRH) agonists (63.79%, 37/58). The rate of recurrence after termination or suppression of ovarian function was 8.33% (7/84), and there was a mortality rate of 1.19% (1/84). Diagnosis of endometriosis-related hemorrhagic ascites may be challenging because it mimics several disease entities that cause ascites, thereby warranting a heightened clinical suspicion. Minimally invasive techniques are usually employed to establish a histologic diagnosis. The prevention of recurrence involves the recognition of endometriosis-related hemorrhagic ascites as a manifestation of severe endometriosis, which should prompt therapies directed at suppressing ovarian function. Since affected women are of childbearing age, ovary-preserving surgeries are generally preferred. The rate of recurrence is low after appropriate surgical and medical interventions.

## Introduction

Hemorrhagic ascites is a rare complication of endometriosis. The first description of endometriosis-related ascites has been attributed to Brews in 1954 [1]. However, it was not until 1957 that Charles first chronicled a case of blood-stained ascites in association with endometriosis [2]. Since then, fewer than 100 reports of hemorrhagic ascites related to endometriosis have been published in the literature.

Endometriosis-related hemorrhagic ascites may manifest with varying symptoms. Recognizing it may be difficult as it may present with similar disease processes such as malignancy, infection, cirrhosis, or trauma [3-6]. In light of this, we conducted this study to examine and elucidate the patient profiles and presentation of the disease to raise clinical awareness among gynecologists regarding the diagnosis of hemorrhagic ascites associated with endometriosis.

## Case presentation

**Figure 1:**
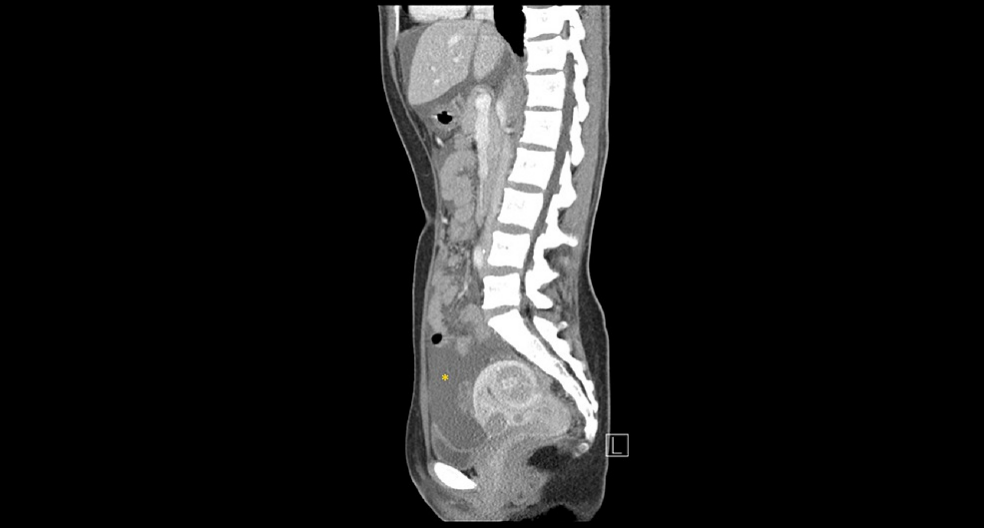
Abdominal CT scan – sagittal view showing massive ascites (asterisk) CT: computed tomography

**Figure 2:**
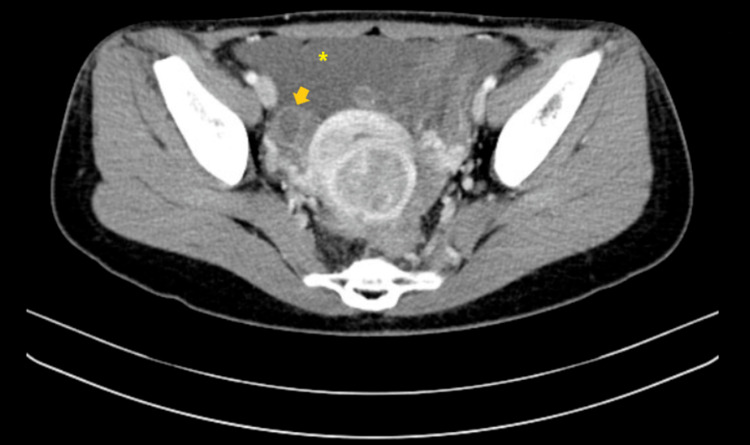
Abdominal CT scan – axial view showing massive ascites (asterisk), right adnexal mass (arrow), and soft tissue seeding CT: computed tomography

A 34-year-old Taiwanese nulligravida woman presented to the outpatient department with a one-year history of irregular dysmenorrhea that was 5/10 in severity. She had no other associated complaints such as weight loss, anorexia, dyspareunia, urinary changes, or heavy menstrual bleeding. On further probing, the patient revealed having mild bloating that did not cause discomfort. Her menstruation occurred at regular monthly intervals. On physical examination, she had clear breath sounds and mildly distended flanks. Pelvic examination showed a corpus enlarged to 8-10 weeks' size without adnexal masses or tenderness. Fullness at the cul-de-sac was palpated. Pelvic ultrasound revealed multiple small leiomyomas with massive ascites and a heterogeneous right ovarian tumor. A CT scan showed a multicystic right ovary with soft tissue seeding to bilateral paracolic gutters, omentum, and recto-uterine pouch, with massive ascites (Figures 1, 2). CA-125 was elevated (819.1 U/mL). With the working diagnosis of a possible malignant ovarian tumor, laparotomy was performed with staging surgery in mind.

**Figure 3:**
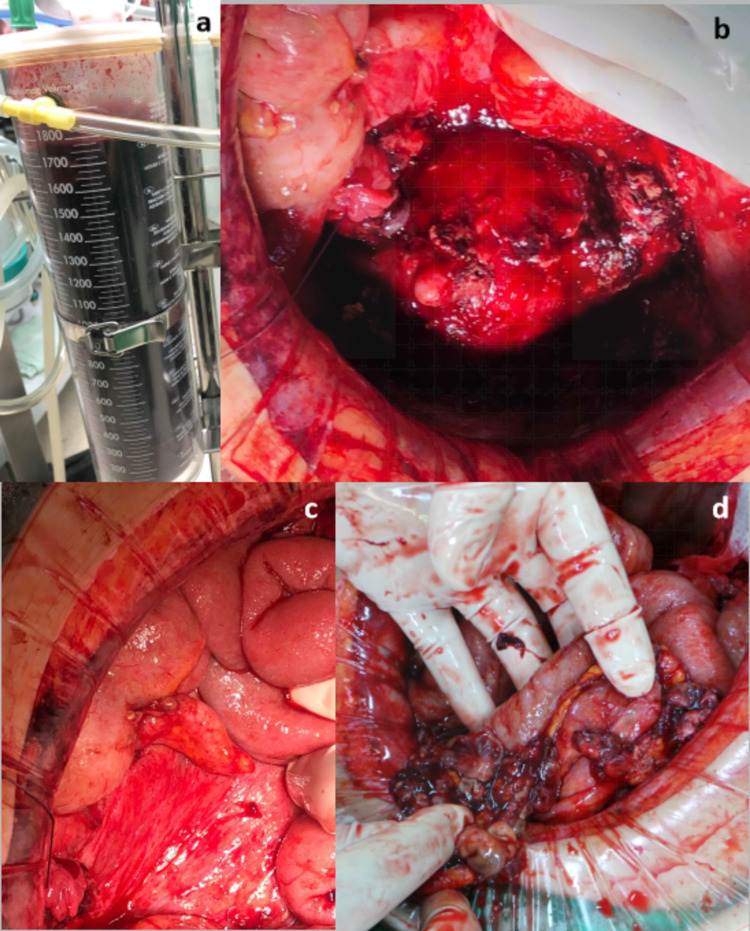
Operative findings a. Hemorrhagic fluid. b. Friable soft tissue lesions on the uterine surface. c. Granular lesions on intestines, soft tissue nodules at the base of the appendix. d. Contracted omentum with numerous gray soft tissue nodules

Intraoperatively, 2 liters of dark-red ascitic fluid was drained (Figure 3a). Both adnexa were plastered to the posterior uterine wall. An ovarian tumor could not be identified. Friable soft tissue lesions were found on the uterine surface (Figure 3b). The cul-de-sac was obliterated. Multiple gray soft tissue nodules were scattered about the contracted omentum, mesentery, and the appendix (Figures 3c, 3d). Minimal manipulation of the pelvic organs provoked bleeding. The frozen section and final histopathological report of the implants were consistent with endometriosis. A diagnosis of stage IV endometriosis was made.

The patient had an uncomplicated postoperative course and was started on leuprorelin injections once a month for six months. After two months, a repeat ultrasound showed mild ascites (~100 mL). The patient remained otherwise asymptomatic on her monthly follow-up visits.

## Discussion

Methods

Literature Search Strategy

An extensive literature search of all case reports, case series, and letters to the editor was performed. PubMed/MEDLINE, Cochrane Library, EMBASE, and Ovid Discovery were searched with the keywords, “endometriosis” OR “endometriotic “OR “endometrioma” AND “ascites” OR “bloody ascites” OR “hemorrhagic ascites” OR “serosanguinous “OR “chocolate” OR “brown fluid” OR “chocolate ascites” OR “brown ascites” OR “serosanguinous ascites”. Human studies involving women with biopsy-proven endometriosis published in any language were included, from inception until December 2018.

Eligibility Criteria

Studies with no available full-texts, non-histologically proven cases of endometriosis, non-hemorrhagic ascites, or those without a description of ascites were excluded. Patients with conditions that may cause ascites or hemorrhage (current tuberculosis, malignancy, other infections, ovulation induction, end-stage renal disease, HIV), history of trauma, pregnancy, were likewise excluded.

Screening and Data Extraction

Two independent reviewers (MCT and WTC) reviewed all titles and abstracts of articles obtained through the online database search. The full-text articles of abstracts that were deemed relevant were retrieved online or by manual searching. Reviewed articles were entered into a standardized data collection matrix. Information on authors, country/continent of origin, year of publication, patient characteristics such as age, parity, and ethnicity were entered into the data matrix. Chief complaint, character and volume of the ascites, interventions, intraoperative findings, severity of endometriosis, and outcomes were likewise recorded. In cases where the exact volume of ascites was not stated in a study, ascites was quantified based on the definitions from the existing literature and consensus reports [7-9]. The severity of endometriosis was recorded in each case or assessed based on intraoperative descriptions vis-a-vis the revised American Society for Reproductive Medicine (ASRM) classification of endometriosis [10].

Quality Assessment of Case Reports

MCT and WTC independently assessed the quality of individual studies based on the checklist for case reports and case series from the Joanna Briggs Institute Critical Appraisal tools for systematic reviews [11].

PRISMA Flow Diagram

**Figure 4:**
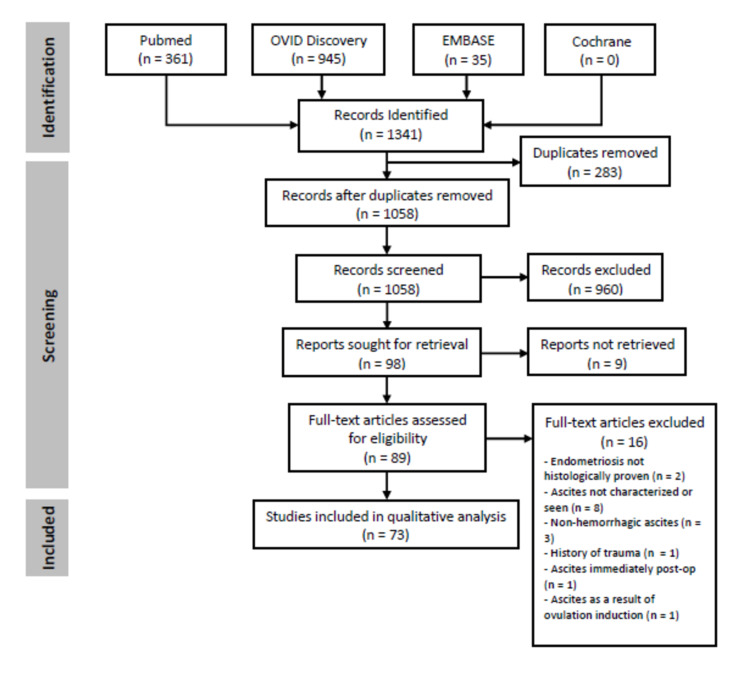
PRISMA flow diagram PRISMA: Preferred Reporting Items for Systematic Reviews and Meta-analyses

The literature search strategy was summarized in a flow diagram based on the protocol laid out by the Preferred Reporting Items for Systematic Reviews and Meta-analyses (PRISMA) Statement [12] (Figure 4).

Statistical Analysis

Descriptive statistics were used to report study and patient characteristics, including symptoms and peritoneal involvement. Spearman rank correlation was used. Analyses were done using the Stata software version 16.0 (StataCorp, College Station, TX).

Results

The literature search initially yielded 1,341 citations for review. After a screening based on the inclusion and exclusion criteria, 73 case reports involving 84 women of endometriosis-related hemorrhagic ascites were included in the final analysis. These were published from 1957 to 2018. The patient demographics, clinical presentation, and management as described in these reports are summarized in Table 1.

**Table 1:** Case reports of endometriosis-related hemorrhagic ascites A: Asian; AFR: of African descent; B: brown/dark brown/brownish/chocolate-colored; BS: bilateral salpingectomy; BSO: bilateral salpingo-oophorectomy; C: Caucasian; COC: combined oral contraceptive pills; coffee: coffee-colored; distension: abdominal distension; DMPA: depot medroxyprogesterone acetate; Dysm: dysmenorrhea; GnRH: gonadotropin-releasing hormone agonists; H: hemorrhagic/bloody; mass: abdominal mass; MPA: medroxyprogesterone acetate; pain: abdominal pain; SS: serosanguinous/blood-stained/haemoserous; TAHBSO: total abdominal hysterectomy with bilateral salpingo-oophorectomy; USO: unilateral salpingo-oophorectomy; RSO: right salpingo-oophorectomy

Study		Patient age (years)	Race	Parity	Chief complaint	CA-125 (U/mL)	Ascites volume (mL)	Ascites color	Pleural effusion	Surgery	Main procedure	Medical management	Recurrence	
														1	Soyman et al., 2018 [13]	31		0	Pain	<35	3000	H	No	Laparotomy	Biopsy	GnRH	No	
														2	Mendes et al., 2018 [14]	31	AFR	0	Distension	192	8500	H	No	Laparoscopy	BS, excision of peritoneum	GnRH, then COC	Yes	
3	Mendes et al., 2018 [14]	26	C	0	Distension	86	≥2000	H	Yes	Laparoscopy	Biopsy	GnRH, then desogestrel	No	
4	Mendes et al., 2018 [14]	37	AFR	0	Distension		5700	H	No	Laparoscopy	Biopsy, excision of nodules	GnRH for 3 months, then desogestrel	No	
5	Walker et al., 2018 [15]	33	A	0	Distension	239	6000	SS	Yes	Laparotomy	Biopsy	GnRH, then dienogest	Yes	
6	O'yandjo et al., 2018 [16]	31	AFR	0	Distension		5000	H	Yes	Laparotomy	Cyst excision	GnRH	No	
7	Magalhães et al., 2018 [17]	28	AFR	0	Weight loss	889.6	8000	H	No	Laparoscopy	Biopsy	GnRH for 6 months	Yes	
8	Petrosellini et al., 2018 [18]	44	AFR	0	Mass	89.8	2000	B	No	Laparotomy	Partial cystectomy	None	No	
9	Pereira et al., 2018 [19]	21		0	Distension		4000	H	No	Laparoscopy	Biopsy	Monophasic COC	Yes	
10	N'Guessan et al., 2017 [20]	26	AFR	0	Distension	63	6000	H	No	Laparoscopy	Biopsy	GnRH, then COC	No	
11	Varun and Tanwar, 2016 [21]	26	A	0	Distension	36.3	3000	H	No	Laparotomy	Cystectomy	GnRH	No	
12	Dun et al., 2016 [22]	26	AFR	0	Distension		7800	H	No	Laparoscopy	Biopsy, peritoneal stripping	None	Yes	
13	Hinduja et al., 2016 [23]	34		1	Distension	<35	4500	SS	No	Laparotomy	TAHBSO	GnRH 250mcg/day for 6 weeks	Yes	
14	Setubal et al., 2015 [24]	26	C	0	Dysm	100	3500	H	No	Laparoscopy	Biopsy	COC	Yes	
15	Bignall et al., 2014 [25]	36	AFR	0	Pain	1123	3500	H	No	Laparoscopy	Biopsy	GnRH + tibolone	Yes	
16	Cosma et al., 2014 [26]	36		0	Dysm	184	4200	B	No	Laparoscopy	Biopsy, excision of all lesions	None	Yes	
17	Hasdemir et al., 2015 [27]	32		0	Distension	41.7	2500	H	Yes	Laparoscopy	Biopsy	GnRH for 6 moths, then dienogest	Yes	
18	Park and Kim, 2014 [28]	44		0	Pain	>10000	≥2000	B	No	Laparotomy	USO, cystectomy	NR	No	
19	Asano et al., 2014 [29]	35	A	0	Dysm	22	5500	H	No	Laparoscopy	Biopsy	GnRH, then dienogest 2 mg PO OD	Yes	
20	Appleby et al., 2014 [30]	34	AFR	0	Distension		4000	H	No	Laparoscopy	Biopsy	GnRH for 6 months	No	
21	Mumtahana et al., 2014 [31]	36	A	0	Distension	5009	3000	H	No	Laparoscopy	Bilateral cystectomy	GnRH	No	
22	Packard and Adamson, 2013 [32]	22	AFR	0	Dyspnea	61	2700	B	Yes	Paracentesis	Biopsy	GnRH, then DMPA	No	
23	Akinola et al., 2012 [33]	26	AFR	0	Cough	72.5	≥1000	H	Yes	Laparotomy	Ovarian mass excision	GnRH 3.6 mg	No	
24	Akintomide et al., 2012 [34]	22	AFR	0	Distension		5900	H	No	Laparotomy	Biopsy	Danazol	Yes	
25	Queirós et al., 2011 [35]	36	C	0	Infertility	73	1500	H	No	Laparoscopy	Cystectomy	COC		
26	Queirós et al., 2011 [35]	30	AFR	0	Infertility	192	12000	B	Yes	Laparoscopy	Biopsy	GnRH, then GnRH + COC		
27	Shabeerali et al., 2012 [36]	40		4	Distension	<35	3000	B	No	Laparoscopy	Biopsy	GnRH for 6 months	Yes	
28	Shabeerali et al., 2012 [36]	30		2	Distension	96	≥1000	B	No	Laparotomy	SubTAH + BSO	None	No	
29	Shabeerali et al., 2012 [36]	28		0	Distension		≥800	H	No	Laparotomy	TAHBSO	None	Yes	
30	Ferrero and Remorgida, 2011 [37]	36			Distension	89.4	4800	H	No	Laparoscopy	Biopsy, excision of nodules	Norethindrone acetate 2.5 mg PO OD	No	
31	Cordeiro Fernandes et al., 2011 [38]	28	AFR	0	Distension	<35	9400	H	No	Laparoscopy	Biopsy	GnRH for 3 months, then COC	No	
32	Suchetha et al., 2010 [39]	36		1	Ascites	>5000	6000	Coffee	No	Laparotomy	TAHBSO	None	No	
33	Ignacio et al., 2010 [40]	38	AFR	0	Distension	50	7000	B	Yes	Laparoscopy	Cystectomy	GnRH + add-back therapy	No	
34	Day et al., 2009 [41]	24		0	Pain		2500	H	No	Laparoscopy	Biopsy	GnRH	Yes	
35	Park et al., 2009 [42]	34		0	Pain	548.1	2000	B	No	Laparoscopy	USO	GnRH + tibolone add-back therapy for 6 months	No	
36	Lodha et al., 2008 [43]	30	AFR	0	Distension		4000	H	No	Laparoscopy	Biopsy	COC	No	
37	Ussia et al., 2008 [44]	23	C	0	Dysm		1500	H	Yes	Laparoscopy	Biopsy	GnRH + intermittent steroids	Yes	
38	Ussia et al., 2008 [44]	26	C	0	Pain		2000	H	No	Laparotomy	USO	GnRH	Yes	
39	Sait, 2008 [45]	26	AFR	0	Distension	3140	5000	H	No	Laparotomy	Bilateral cystectomy	GnRH for 6 months, then COC	No	
40	Santos et al., 2007 [46]	40	C	0	Pain		≥2000	SS	No	Laparotomy	Biopsy	None, mortality	No	
41	Palayekar et al., 2007 [47]		AFR	1	Distension	33.6	4000-6000	H	No	Laparotomy	TAHBSO	None	No	
42	Goumenou et al., 2006 [3]	46	C	0	Dyspnea	3504	4000	H	Yes	Laparotomy	TAHBSO	None	No	
43	Baykal et al., 2006 [48]	30		0	Distension	2540	≥1000	B	No	Laparotomy	USO	NR	No	
44	Ekoukou et al., 2005 [49]	28	AFR	0	Infertility		10000	H	No	Laparoscopy	Biopsy	GnRH	Yes	
45	Fortier et al., 2005 [50]	33	AFR	0	Infertility	257	4000	SS	Yes	Laparoscopy	Cystectomy	GnRH	Yes	
46	Zeppa et al., 2004 [51]	34					500	H	No	Paracentesis	Paracentesis	NR	No	
47	Francis et al., 2003 [52]			2	Dyspnea	<35	≥2000	B	Yes	Laparotomy	TAHBSO	None	No	
48	Cheong and Lim, 2003 [53]	40	A	1	Distension	<35	5600	H	Yes	Laparotomy	Biopsy	NR	No	
49	Moffatt and Mitchell, 2002 [54]	37	AFR	0	Dyspnea	<35	≥2000	B	Yes	Laparotomy	TAHBSO	GnRH	Yes	
50	Dias et al., 2000 [55]	41	AFR	0	Distension		10000	B	No	Laparotomy	USO	GnRH for 6 months	Yes	
51	Bhojawala et al., 2000 [56]	34	AFR	0	Distension		9000	B	Yes	Laparotomy	TAHUSO	None	No	
52	El Khalil et al., 1999 [57]	36			Distension		3500	H	No	Laparoscopy	Biopsy	COC	Yes	
53	Samora-Mata and Feste, 1999 [58]	43	C	3	Pain		2000	B	No	Laparotomy	TAHRSO	None	No	
54	Fletcher et al., 1999 [59]	27	AFR	1	Distension		8000	B	No	Laparotomy	Biopsy	GnRH monthly for 6 months	No	
55	Muneyyirci-Delale et al., 1998 [60]	26	AFR		Pain	455	2000	H	Yes	Laparotomy	Bilateral cystectomy	Danazol 600 mg PO daily for 6 months, then norethindrone acetate	Yes	
56	Muneyyirci-Delale et al., 1998 [60]	31	AFR	0	Shortness of breath		10000	B	Yes	Laparotomy	TAHBSO	None	Yes	
57	Muneyyirci-Delale et al., 1998 [60]	32	AFR	0	Distension		4900	H	No	Laparotomy	Ovarian wedge resection	GnRH	No	
58	Muneyyirci-Delale et al., 1998 [60]	35	AFR	1	Dysm	266	3000	H	No	Laparotomy	Adnexal mass resection	GnRH for 6 months, then norethindrone acetate	No	
59	Mejia et al., 1997 [61]	44	AFR	0	Distension	<35	10000	H	No	Laparotomy	TAHBSO	None	No	
60	Flanagan and Barnes, 1996 [62]	30	AFR		Distension	49	2000	B	Yes	Laparotomy	USO, ovarian wedge resection	GnRH	Yes	
61	el-Newihi et al., 1995 [63]	32	AFR	0	Distension	118	4000	B	Yes	Laparotomy	TAHBSO	GnRH IM monthly for 6 months	No	
62	Schlueter and McClennan, 1994 [64]	20	AFR	0	Distension		5000	H	No	Laparoscopy	Biopsy	GnRH monthly	No	
63	Jose et al., 1994 [65]	30		0	Distension		5000	B	Yes	Laparotomy	USO	Danazol 200 mg TID	No	
64	London and Parmley, 1993 [66]	29	AFR	0	Distension		3000	B	No	Laparotomy	TAHBSO	None	No	
65	Chen et al., 1992 [67]	20	A	0	Distension	46	5600	B	Yes	Laparotomy	USO	Danazol 400 mg PO daily + Duphaston 10 mg PO OD for 6 months	No	
66	Tsvelev et al., 1990 [68]	31			Pain		8000	B	No	Laparotomy	USO	NR	No	
67	Yu and Grimes, 1991 [69]	26	A	0	Pain		3000	H	Yes	Laparotomy	USO	GnRH for 6 months	No	
68	Hattori et al., 1990 [70]	50	A	2	Distension	36	3800	B	No	Laparotomy	TAHBSO	MPA	Yes	
69	Taub et al., 1989 [6]	32	AFR	1	Distension		3400	H	Yes	Laparotomy	BSO	DMPA	No	
70	Olubuyide et al., 1988 [71]	19	AFR	0	Distension		4600	H	No	Laparotomy	Biopsy	Norethisterone acetate 5 mg PO TID for 1 week, then 10 mg BID	No	
71	Chichareon and Wattanakitkrailert, 1988 [72]	31		0	Distension		1800	H	No	Laparotomy	TAHUSO	DMPA	Yes	
72	Iwasaka et al., 1985 [73]	35	A	0	Distension	17	2500	B	No	Laparotomy	TAHBSO	None	No	
73	Iwasaka et al., 1985 [73]	25	A	0	Pain		150	H	No	Laparotomy	USO, Ovarian wedge resection	Danazol 400 mg PO daily for 3 months	No	
74	Naraynsingh et al., 1985 [74]	24	AFR	0	Distension		6000	H	No	Laparotomy	Biopsy	DMPA IM q2 weeks for 6 months	No	
75	Halme et al., 1985 [75]	23	AFR	0	Distension		7500	SS	No	Laparotomy	Biopsy	Danazol 400 mg PO BID	No	
76	Jenks et al., 1984 [76]	33	AFR	0	Distension		5000	H	No	Laparotomy	TAHBSO	None	No	
77	Gaulier et al., 1983 [77]	22	AFR	0	Pain		≥2000	B	Yes	Laparotomy	Ovarian resection	Danazol	No	
78	Chervenak et al., 1981 [78]	20		0	Distension		1500	B	No	Laparotomy	BSO	None	No	
79	Chervenak et al., 1981 [78]	26	AFR	0	Distension		4000	B	No	Laparotomy	BSO	Danazol 400 mg daily for 10 months	No	
80	Irani et al., 1976 [79]	32	AFR	0	Distension		2000	H	Yes	Laparotomy	TAHBSO	None	No	
81	Collier et al., 1962 [80]	34	AFR	0	Distension		4000	B	No	Laparotomy	TAHBSO	None	Yes	
82	Bernstein et al., 1961 [81]	29	AFR	1	Distension		3900	B	No	Laparotomy	TAHBSO	None	No	
83	Ripstein et al., 1959 [82]	24	AFR	0	Chest discomfort		100-150	B	Yes	Laparotomy	Biopsy	COC	No	
84	Charles, 1957 [2]	33		0	Pain		3000	H	Yes	Laparotomy	USO	Deep X-ray therapy	Yes	

Patient characteristics are shown in Table 2. The mean age of the patients at diagnosis was 31.16 years (SD: 6.57; range: 19-50). There was no relationship between the year of publication/presentation and age (p=0.193) or age distribution (p=0.600).

**Table 2:** Endometriosis-related hemorrhagic ascites – patient characteristics SD: standard deviation

Characteristics	Values
Age, years, mean (SD)	31.16 (6.57)
Age range, years	19-50
Age distribution, number (%), N=82	
<20 years	1 (1.22)
20-29 years	31 (37.80)
30-39 years	40 (48.78)
40-49 years	9 (10.98)
≥50 years	1 (1.22)
Parity, number (%), N=78	
Nulliparous	65 (83.33)
Parous	13 (16.67)
Race distribution, number (%), n=62	
African	43 (69.35)
Asian	10 (16.13)
Caucasian	9 (14.52)
Ascitic fluid volume, mL, mean (SD)	4228.27 (2625.66)

The most common presenting symptom was abdominal distension (Table 1). Other initial complaints reported by patients are presented in Table 3. The majority (91.67%, 77/84) of the symptoms were gradual in onset. Pleural effusion was reported in 32.14% (27/84) of cases. The ascitic fluid was predominantly massive with a mean volume of 4228.27 mL (SD: 2625.66; range: 100-10000). CA-125 was elevated in 32 out of 43 patients, with a median value of 86 U/mL (range: 17->10000 U/mL).

**Table 3:** Symptoms of hemorrhagic ascites associated with endometriosis (N=84)

Symptom	Number (%)
Abdominal distension	66 (78.57)
Dysmenorrhea	47 (55.95)
Abdominal pain	28 (33.33)
Weight loss	18 (21.43)
Primary infertility	17 (20.24)
Nausea and/or vomiting	13 (15.48)
Anorexia	11 (13.10)
Dyspnea	9 (10.71)
Deep dyspareunia	6 (7.14)
Fatigue/malaise	6 (7.14)
Chronic pelvic pain	5 (5.95)
Constipation	5 (5.95)
Shortness of breath	4 (4.76)
Early satiety	4 (4.76)
Cough	3 (4.57)
Dyschezia	3 (3.57)
Menorrhagia	3 (3.57)
Right-sided chest discomfort	3 (3.57)
Weight gain	2 (2.38)
Loose stools	2 (2.38)
Dysuria	2 (2.38)
Orthopnea	1 (1.19)
Abdominal mass	1 (1.19)
Thoracic pain	1 (1.19)

Moderate to severe endometriosis (ASRM stage III to IV) was seen in 97.44% (76/78) of the cases, and adhesions were described in 78.05% (64/82). In 43.90% (36/82) of the cases, an ovarian cyst was identified; 11.11% (4/36) of the cases were ruptured. Peritoneal implants scattered about the abdominopelvic cavity in 42.68% (35/82), while peritoneal nodules were seen in 20/82 (24.39%). Other abdominopelvic areas involved are shown in Table 4.

**Table 4:** Peritoneal involvement in endometriosis-related hemorrhagic ascites (N=82)

Organ involved	Number (%)
Intestines	52 (63.41)
Recto-sigmoid	27 (32.93)
Omentum (caking/nodule/retraction/implants)	25 (30.49)
Cul-de-sac	23 (28.05)
Liver	10 (12.20)
Diaphragm	7 (8.54)
Appendix	6 (7.32)
Rectovaginal area	5 (6.10)
Umbilicus (nodule/mass/cyst)	4 (4.88)

At the time of presentation, 64.29% (54/84) underwent laparotomy, and laparoscopy was performed in 33.33% (28/84). Two cases (2/84) had paracentesis. Almost half (44.05%, 37/84) of the cases had repeat abdominal surgeries, while 76.19% (64/84) required multiple procedures that included repeat abdominal surgeries (laparoscopy and/or laparotomy), paracentesis, thoracostomy, or thoracotomy. On the other hand, less invasive surgical approaches (p<0.001) and fertility-sparing procedures (p<0.001) are observed to be increasingly favored in recent years.

A cure was reported in 95.45% (21/22) who went through definitive surgery via hysterectomy with bilateral salpingo-oophorectomy. Medical treatment was not given to 68.18% (15/22) after surgery. Four patients tolerated stripping or excision of the peritoneum of all endometriotic implants with no recurrence. Two of these received no additional medical therapy.

Patients who were offered medical therapy post-surgery received gonadotropin-releasing hormone (GnRH) agonists (63.79%, 37/58), either alone, with add-back therapy, or as a preliminary treatment that was eventually transitioned to either a progestogen or a combined oral contraceptive (COC) pill. In 86.49% (32/37) who received GnRH agonists, no recurrences were observed. Other therapies included danazol (13.79%, 8/58), progestogens alone (10.34%, 6/58), or COC alone (10.34%, 6/58). The cure rate with danazol was 100% (eight out of eight), while COC and progestogens were equally effective, each with an 83.33% (five out of six) cure rate.

The recurrence rate observed at the time of presentation or after initial management was 36.90% (31/84), while that after definitive surgery and/or ovarian function suppression was 8.33% (7/84). Five of these cases reported significant ascites upon the cessation of GnRH therapy [35,49,50,62] or upon shifting from GnRH to progestogen therapy [15]. The other two had reaccumulating minimal ascites while on oral COC [35] or oral progestogen [70]. Of note, 71.42% (five out of seven) of recurrences had undergone ovary-preserving procedures (oophorocystectomy or biopsy) prior to medical therapy. Mortality was reported in one case. The Median follow-up period was eight months.

Analysis

Very little is known about the pathogenesis of endometriosis-related hemorrhagic ascites. One putative mechanism is peritoneal irritation from the rupture of ovarian cysts. The endometrial cells from this spillage propagate the spread of implants in the pelvic cavity and cause inflammation, which in turn leads to adhesions and ascites [81]. This theory assumes the presence of ovarian cysts. However, in this review, less than half of the study population were found to have ovarian endometriotic cysts, and only four out of 36 of these cysts were ruptured. Alternative hypotheses such as alterations in vascular permeability, lymphatic channel obstruction, as well as individual variations in susceptibility to the disease may be explored [44,49,83,84].

The rubor of ascites may be due to increased angiogenesis seen in endometriosis. Erosions from affected friable soft tissue, serosal, peritoneal surfaces, and implants cause micro-bleeding or frank bleeding, leading to the hemorrhagic character of ascites [49,84]. Pleural effusions associated with the hemorrhagic ascites may be due to several mechanisms. However, based on the presentation of massive ascites in the majority of cases, the most plausible cause is anatomic defects in the diaphragm that allow for the passage of hemorrhagic fluid into the pleural space [85,86].

Endometriosis-related hemorrhagic ascites may affect any woman of reproductive age but is more common in women in their twenties and thirties, without any significant increase or decrease with respect to the age of onset. This finding differs from what was previously described [44]. Many patients may seek a consult for abdominal distension or symptoms secondary to abdominal distension such as pain or pulmonary discomfort in the background of dysmenorrhea or worsening dysmenorrhea. Dysmenorrhea accounted for only 5.95% (5/84) of the chief complaints in this review but is most commonly elicited on history as an accompanying symptom. Massive ascites usually predominate in clinical evaluation.

The utility of CA-125 in the diagnosis of this condition is arguable due to its non-specificity. While the majority presented with CA-125 >35 U/mL, similarly increased levels have been described in various benign gynecologic diseases [87]. Mesothelial cells that line the peritoneum secrete CA-125. Since mesothelial hyperplasia and hypertrophy are associated with endometriosis, CA-125 release is greater, and hence elevated in this condition. However, the same holds true for other diseases of the peritoneum such as malignancy and tuberculosis [84,88,89]. Its clinical use, therefore, is limited to determining whether a patient has peritoneal disease in general.

Management of the condition relies critically on establishing a histologic diagnosis. Surgery is thus warranted, although several studies have achieved cytological confirmation through paracentesis [32,51]. With the case presented, a clinically presumptive diagnosis of ovarian cancer was made, which led to the decision to perform a laparotomy. This is supported by studies on ovarian cancer [90]. However, with the availability of minimally invasive techniques and increasing technical confidence among surgeons, there is a growing trend favoring their use in the management of potentially malignant ovarian tumors [90,91]. The current recommendation for laparoscopy in suspected ovarian tumors is to establish a histologic diagnosis through a frozen section and, if tumors are found malignant, to assess their resectability [91-93]. Since it is difficult to differentiate it from a malignant etiology, surgical management of endometriosis-related hemorrhagic ascites may follow this approach.

Moderate to severe (ASRM stage III to IV) endometriosis almost always presents intraoperatively and with adhesions and implants in the abdominopelvic cavity. Peritoneal involvement can be related to small implants, nodules, or varying degrees of adhesions. Thus, the presence of hemorrhagic ascites, as seen in 97.44% of cases and in the index case, may correlate with the severity of endometriosis.

Since the ascites in this review was found mostly in moderate to severe endometriosis, it seems logical to follow the principles of endometriosis treatment. Termination or suppression of ovarian function is the cornerstone of management. The importance of this cannot be overemphasized as many women undergo multiple surgeries for recurrence or for the treatment of an existing endometriosis. Surgical sterility via hysterectomy with removal of bilateral ovaries is the definitive form of management [19,36 59,61,63,68]. However, fertility-sparing surgeries are currently performed in patients who wish to realize their reproductive potential.

Medical therapy consists of GnRH agonists, which have been used with success in achieving ovarian suppression. Danazol, progestogens, and COC pills are likewise given as primary treatment or upon completion of GnRH agonist therapy for long-term control of the disease. Danazol, an antigonadotropic, anti-estrogenic synthetic steroid, is effective in suppressing ovarian function. However, its various androgenic effects preclude its use [94,95]. In the majority of cases and especially in more recent studies, GnRH agonists have been used more frequently. These are effective in achieving ovarian suppression and increasing fertility rates but their side effect profile limits their long-term use [94,95]. Progestogens and COC pills were effective as medical treatments in this review, but current evidence has failed to demonstrate any benefit of COC in managing pelvic pain in endometriosis [96]. On the other hand, oral medroxyprogesterone acetate has been shown to be effective in decreasing chronic pelvic pain [97]. Other medications of interest are the levonorgestrel-releasing intrauterine system and mifepristone, which were not used in the studies included in this review. Nonetheless, their clinical utility may be explored as these have been shown to be effective in suppressing the menstrual cycles and relieving pain associated with endometriosis [98,99].

## Conclusions

Hemorrhagic ascites is a rare manifestation of endometriosis that can present in any premenopausal woman. The most common initial complaint is abdominal distension, but a host of other symptoms may also be associated with the condition. Diagnosis can be challenging because it mimics several disease entities that cause ascites, thus warranting a heightened clinical suspicion. Minimally invasive techniques may be employed to establish a histologic diagnosis. Recognition of hemorrhagic ascites as a manifestation of severe endometriosis is essential for recurrence prevention, which should prompt therapies directed at suppressing ovarian function. Ovary-preserving surgeries are preferred because affected women are of childbearing age. Recurrence is low after appropriate surgical and medical interventions.

## References

[1] Brews A (1954). Endometriosis including endometriosis of the diaphragm and Meigs' syndrome. Proc R Soc Med.

[2] Charles D (1957). Endometriosis and hemorrhagic pleural effusion. Obstet Gynecol.

[3] Goumenou A, Matalliotakis I, Mahutte N, Koumantakis E (2006). Endometriosis mimicking advanced ovarian cancer. Fertil Steril.

[4] Myers TJ, Arena B, Granai CO (1995). Pelvic endometriosis mimicking advanced ovarian cancer: presentation with pleural effusion, ascites, and elevated serum CA 125 level. Am J Obstet Gynecol.

[5] Urrunaga NH, Singal AG, Cuthbert JA, Rockey DC (2013). Hemorrhagic ascites. Clinical presentation and outcomes in patients with cirrhosis. J Hepatol.

[6] Taub WH, Rosado S, Kalaycioglu M, Booher D, Barnes DS (1989). Hemorrhagic ascites secondary to endometriosis. J Clin Gastroenterol.

[7] Goldberg BB, Goodman GA, Clearfield HR (1970). Evaluation of ascites by ultrasound. Radiology.

[8] Moore KP, Wong F, Gines P (2003). The management of ascites in cirrhosis: report on the consensus conference of the International Ascites Club. Hepatology.

[9] Moore CM, Van Thiel DH (2013). Cirrhotic ascites review: pathophysiology, diagnosis and management. World J Hepatol.

[10] No authors listed (1997). Revised American Society for Reproductive Medicine classification of endometriosis: 1996. Fertil Steril.

[11] Moola S, Munn Z, Tufanaru C (2019). Systematic reviews of etiology and risk. JBI Manual for Evidence Synthesis.

[12] Page MJ, McKenzie JE, Bossuyt PM (2021). The PRISMA 2020 statement: an updated guideline for reporting systematic reviews. BMJ.

[13] Soyman Z, Bacanakgil BH, Kaya S (2018). An uncommon presentation of endometriosis a case report. J Reprod Med.

[14] Mendes S, Carvalho C, Rodrigues G, Barata S, Calhaz-Jorge C, Osório F (2018). Successful treatment of endometriosis-related hemorrhagic ascites: a report of three cases. Surg Technol Int.

[15] Walker PJ, Johnson NP (2018). Benign endometriosis masquerading as intra-abdominal malignancy: one of the most extreme cases reported and a review of the literature. J Endometr Pelvic Pain Disord.

[16] O’yandjo AM, Bosenge NJD, Kadima NJ (2018). Umbilical nodule and hemorrhagic ascites of endometriosis origin: a clinical case report. Gynecol Obstet Case Rep.

[17] Magalhães TF, Augusto KL, Mota LP, Costa ARD, Puster RA, Bezerra LRPS (2018). Ascites and encapsulating peritonitis in endometriosis: a systematic review with a case report. Rev Bras Ginecol Obstet.

[18] Petrosellini C, Abdalla S, Oke T (2018). The many guises of endometriosis: giant abdominal wall endometriosis masquerading as an incisional hernia. Int J Fertil Steril.

[19] Pereira N, Gunnala V, Palermo GD, Elias RT (2018). Laparoscopic management of severe endometriosis-related hemorrhagic ascites. J Minim Invasive Gynecol.

[20] N’Guessan E, Kouamé N, Dia JM, Gbeli F, Guié P, Anongba S (2017). Endometriosis revealed by recurrent hemorrhagic ascites. Open J Obstet Gynecol.

[21] Varun N, Tanwar RT (2016). A rare presentation of endometriosis with massive haemorrhagic ascites: a case report. Gynecol Obstet Case Rep.

[22] Dun EC, Wong S, Lakhi NA, Nehzat CH (2016). Recurrent massive ascites due to mossy endometriosis. Fertil Steril.

[23] Hinduja I, Kapadia K, Udwadia F, Bhilawadikar R, Adhe A, Zaveri K (2016). Unusual presentation of endometriosis with haemorrhagic ascites - a case report. J Obstet Gynaecol.

[24] Setubal A, Sidiropoulou Z, Soares S, Barbosa C (2015). Endometriosis and ascites: a strategy to achieve pregnancy. J Minim Invasive Gynecol.

[25] Bignall J, Arambage K, Vimplis S (2014). Endometriosis: a rare and interesting cause of recurrent haemorrhagic ascites. BMJ Case Rep.

[26] Cosma S, Ceccaroni M, Benedetto C (2014). A pseudoneoplastic finding of deep endometriosis: laparoscopic triple segmental bowel resection. Wideochir Inne Tech Maloinwazyjne.

[27] Hasdemir PS, Ikiz N, Ozcakir HT, Kara E, Guvenal T (2015). Endometriosis associated with relapsing ascites and pleural effusions. J Obstet Gynaecol.

[28] Park CM, Kim SY (2014). Rupture of an endometrioma with extremely high serum CA-125 level (> 10,000 IU/ml) and ascites resembling ovarian cancer. Eur J Gynaecol Oncol.

[29] Asano R, Nakazawa T, Hirahara F, Sakakibara H (2014). Dienogest was effective in treating hemorrhagic ascites caused by endometriosis: a case report. J Minim Invasive Gynecol.

[30] Appleby R, Saroya H, Postgate A, Meer Z (2014). A young woman with abdominal distension. BMJ Case Rep.

[31] Mumtahana F, Jiao J, Cui B (2014). A rare presentation of endometriosis with recurrent massive hemorrhagic ascites which can mislead. Int J Women’s Health Reproduction Sci.

[32] Packard LK, Adamson GD (2013). Endometriosis presenting with massive ascites and pleural effusion: a case report. J Endometr Pelvic Pain Disord.

[33] Akinola RA, Akinola OI, Alakija A, Wright KO (2012). Widespread endometriosis mimicking ovarian malignancy: a case report. Niger Postgrad Med J.

[34] Akintomide AO, Bassey DB, Ekanem EI, Omotosho AJ (2012). Omental endometriosis: a rare site and an unusual association with ovarian fibroma and haemorrhagic ascites. A case report and review of the imaging techniques. IOSR JDMS.

[35] Queirós A, Correia L, Pinto G, Rosa D, Silva G, Simões T (2011). Endometriosis with hemorrhagic ascites: two cases report (Article in Portuguese). Rev Iberoam Fert Rep Hum.

[36] Shabeerali TU, Rajan R, Kuruvilla AP (2012). Hemorrhagic ascites: are we missing endometriosis?. Indian J Gastroenterol.

[37] Ferrero S, Remorgida V (2011). Endometriosis presenting with hemorrhagic ascites. Arch Gynecol Obstet.

[38] Cordeiro Fernandes LF, Podgaec S, Castro Cotti GC, Abrao MS (2011). Severe endometriosis may be considered in the differential diagnosis in young women presenting massive hemorrhagic ascites. Gynecol Surg.

[39] Suchetha S, Rema P, Mathew AP, Sebastian P (2010). Endometriosis with massive hemorrhagic ascites. Indian J Cancer.

[40] Ignacio MM, Joseph N, Hélder F, Mamourou K, Arnaud W (2010). Massive ascites, pleural effusion, and diaphragmatic implants in a patient with endometriosis. Eur J Obstet Gynecol Reprod Biol.

[41] Day T, Hui K, Perkins S, Pelletier P (2009). Ascites and ileus due to endometriosis. J Pelvic Med Surg.

[42] Park BJ, Kim TE, Kim YW (2009). Massive peritoneal fluid and markedly elevated serum CA125 and CA19-9 levels associated with an ovarian endometrioma. J Obstet Gynaecol Res.

[43] Lodha A, Klein T, Elish D, Tarkovsky R (2008). Endometriosis: a rare presentation as hemorrhagic ascites. Pract Gastroenterol.

[44] Ussia A, Betsas G, Corona R, De Cicco C, Koninckx PR (2008). Pathophysiology of cyclic hemorrhagic ascites and endometriosis. J Minim Invasive Gynecol.

[45] Sait KH (2008). Massive ascites as a presentation in a young woman with endometriosis: a case report. Fertil Steril.

[46] Santos VM, Barbosa ER Jr, Lima SH, Porto AS (2007). Abdominal cocoon associated with endometriosis. Singapore Med J.

[47] Palayekar M, Jenci J, Carlson JA Jr (2007). Recurrent hemorrhagic ascites: a rare presentation of endometriosis. Obstet Gynecol.

[48] Baykal C, Arioglu P, Kalayci M, Özkan F, Çetinkaya N, Fiçicioglu C (2006). Giant endometrioma mimicking ovarian carcinoma: case report. Turk J Obstet Gynecol.

[49] Ekoukou D, Guilherme R, Desligneres S, Rotten D (2005). Endometriosis with massive hemorrhagic ascites: a case report and review of the literature (Article in French). J Gynecol Obstet Biol Reprod (Paris).

[50] Fortier D, Dedecker F, Gabriele M, Graesslin O, Barau G (2005). Endometriosis with ascites and pleural effusion: a case report (Article in French). Gynecol Obstet Fertil.

[51] Zeppa P, Vetrani A, Cozzolino I, Palombini L (2004). Endometrial glands in ascites secondary to endometriosis. Diagn Cytopathol.

[52] Francis M, Badero OO, Borowsky M, Lee YC, Abulafia O (2003). Pericardial effusion, right-sided pleural effusion and ascites associated with stage IV endometriosis. A case report. J Reprod Med.

[53] Cheong EC, Lim DT (2003). Massive ascites--an uncommon presentation of endometriosis. Singapore Med J.

[54] Moffatt SD, Mitchell JD (2002). Massive pleural endometriosis. Eur J Cardiothorac Surg.

[55] Dias CC, Andrade JM, Ferriani RA, Villanova MG, Meirelles RS (2000). Hemorrhagic ascites associated with endometriosis. A case report. J Reprod Med.

[56] Bhojawala J, Heller DS, Cracchiolo B, Sama J (2000). Endometriosis presenting as bloody pleural effusion and ascites-report of a case and review of the literature. Arch Gynecol Obstet.

[57] El Khalil T, Mourad FH, Barada K, Uthman S (1999). Massive hemorrhagic ascites secondary to endometriosis. J Clin Gastroenterol.

[58] Samora-Mata J, Feste JR (1999). Endometriosis ascites: a case report. JSLS.

[59] Fletcher H, McFarlane M, Shirley SE, Clarke WF, Lyon K (1999). Massive ascites secondary to severe endometriosis. West Indian Med J.

[60] Muneyyirci-Delale O, Neil G, Serur E, Gordon D, Maiman M, Sedlis A (1998). Endometriosis with massive ascites. Gynecol Oncol.

[61] Mejia EM, Alvarez OA, Lee M (1997). Endometriosis with massive bloody ascites. J Am Board Fam Pract.

[62] Flanagan KL, Barnes NC (1996). Pleural fluid accumulation due to intra-abdominal endometriosis: a case report and review of the literature. Thorax.

[63] el-Newihi HM, Antaki JP, Rajan S, Reynolds TB (1995). Large bloody ascites in association with pelvic endometriosis: case report and literature review. Am J Gastroenterol.

[64] Schlueter FJ, McClennan BL (1994). Massive ascites and pleural effusions associated with endometriosis. Abdom Imaging.

[65] Jose R, George SS, Seshadri L (1994). Massive ascites associated with endometriosis. Int J Gynaecol Obstet.

[66] London S, Parmley T (1993). Endometriosis and ascites. South Med J.

[67] Chen FF, Chow NH, Chou CY, Lin MF (1992). Hemorrhagic ascites associated with endometriosis: a rare clinical presentation. J Gynecol Surg.

[68] Tsvelev IuV, Lishchuk VD, Kolosov AE (1990). Ascites as a manifestation of generalized endometriosis (Article in Russian). Vestn Khir Im I I Grek.

[69] Yu J, Grimes DA (1991). Ascites and pleural effusions associated with endometriosis. Obstet Gynecol.

[70] Hattori S, Tamakoshi K, Oguchi H, Kodama H (1990). A case of endometriosis with massive ascites (Article in Japanese). Nihon Sanka Fujinka Gakkai Zasshi.

[71] Olubuyide IO, Adebajo AO, Adeleye JA, Solanke TF (1988). Massive ascites associated with endometriosis in a Nigerian African. Int J Gynaecol Obstet.

[72] Chichareon SB, Wattanakitkrailert S (1988). Endometriosis with ascites. Acta Obstet Gynecol Scand.

[73] Iwasaka T, Okuma Y, Yoshimura T, Kidera Y, Sugimori H (1985). Endometriosis associated with ascites. Obstet Gynecol.

[74] Naraynsingh V, Raju GC, Ratan P, Wong J (1985). Massive ascites due to omental endometriosis. Postgrad Med J.

[75] Halme J, Chafe W, Currie JL (1985). Endometriosis with massive ascites. Obstet Gynecol.

[76] Jenks JE, Artman LE, Hoskins WJ, Miremadi AK (1984). Endometriosis with ascites. Obstet Gynecol.

[77] Gaulier A, Jouret-Mourin A, Marsan C (1983). Peritoneal endometriosis. Report of a case with cytologic, cytochemical and histopathologic study. Acta Cytol.

[78] Chervenak FA, Greenlee RM, Lewenstein L, Tovell HM (1981). Massive ascites associated with endometriosis. Obstet Gynecol.

[79] Irani S, Atkinson L, Cabaniss C, Danovitch SH (1976). Pleuroperitoneal endometriosis. Obstet Gynecol.

[80] Collier HA, Gonzales LL, Bossert LJ (1962). Cyclic ascites as a manifestation of endometriosis. Report of a case. Obstet Gynecol.

[81] Bernstein JS, Perlow V, Brenner JJ (1961). Massive ascites due to endometriosis. Am J Digest Dis.

[82] Ripstein CB, Robman M, Wallach JB (1959). Endometriosis involving the pleura. J Thorac Surg.

[83] Koninckx PR, Renaer M, Brosens IA (1980). Origin of peritoneal fluid in women: an ovarian exudation product. Br J Obstet Gynaecol.

[84] Koninckx PR, Kennedy SH, Barlow DH (1999). Pathogenesis of endometriosis: the role of peritoneal fluid. Gynecol Obstet Invest.

[85] Hwang SM, Lee CW, Lee BS, Park JH (2015). Clinical features of thoracic endometriosis: a single center analysis. Obstet Gynecol Sci.

[86] Nwiloh J (2011). Diaphragmatic patch: a useful adjunct in surgical treatment of recurrent catamenial hemothorax. Rev Port Pneumol.

[87] Daoud E, Bodor G (1991). CA-125 concentrations in malignant and nonmalignant disease. Clin Chem.

[88] Chen DX, Schwartz PE, Li XG, Yang Z (1988). Evaluation of CA 125 levels in differentiating malignant from benign tumors in patients with pelvic masses. Obstet Gynecol.

[89] Oparka R, McCluggage WG, Herrington CS (2011). Peritoneal mesothelial hyperplasia associated with gynaecological disease: a potential diagnostic pitfall that is commonly associated with endometriosis. J Clin Pathol.

[90] Rimbach S, Neis K, Solomayer E, Ulrich U, Wallwiener D (2014). Current and future status of laparoscopy in gynecologic oncology. Geburtshilfe Frauenheilkd.

[91] Angeles MA, Martínez-Gómez C, Migliorelli F (2018). Novel surgical strategies in the treatment of gynecological malignancies. Curr Treat Options Oncol.

[92] Ratnavelu ND, Brown AP, Mallett S (2016). Intraoperative frozen section analysis for the diagnosis of early stage ovarian cancer in suspicious pelvic masses. Cochrane Database Syst Rev.

[93] Morton R, Anderson L, Carter J, Pather S, Saidi SA (2017). Intraoperative frozen section of ovarian tumors: a 6-year review of performance and potential pitfalls in an Australian tertiary referral center. Int J Gynecol Cancer.

[94] Brown J, Farquhar C (2014). Endometriosis: an overview of Cochrane Reviews. Cochrane Database Syst Rev.

[95] Berlanda N, Somigliana E, Viganò P, Vercellini P (2016). Safety of medical treatments for endometriosis. Expert Opin Drug Saf.

[96] Brown J, Crawford TJ, Datta S, Prentice A (2018). Oral contraceptives for pain associated with endometriosis. Cochrane Database Syst Rev.

[97] Brown J, Kives S, Akhtar M (2012). Progestagens and anti-progestagens for pain associated with endometriosis. Cochrane Database Syst Rev.

[98] Fu J, Song H, Zhou M, Zhu H, Wang Y, Chen H, Huang W (2017). Progesterone receptor modulators for endometriosis. Cochrane Database Syst Rev.

[99] Abou-Setta AM, Houston B, Al-Inany HG, Farquhar C (2013). Levonorgestrel-releasing intrauterine device (LNG-IUD) for symptomatic endometriosis following surgery. Cochrane Database Syst Rev.

